# Supplementary material for: A Likelihood Approach for Real-Time Calibration of Stochastic Compartmental Epidemic Models
Source: PLoS Comput Biol. 2017 Jan 17;13(1):e1005257. doi: 10.1371/journal.pcbi.1005257 (PMC5240920; doi:10.1371/journal.pcbi.1005257)
Supplement: S1 File — (TAR.GZ) [file pcbi.1005257.s014.tar.gz › HSPH_Online-SI-Revision/MSS10/n1-mild/n1-mild_NEW_table0.pdf]

1	R0	{1.27677, 7.89375, 1.}	{1.41407, 8.62266, 0.38}	{{1.39882, 8.26611, 0.}, {1.42801, 9.02643, 1.}}	{10.7539, 9.53213, 62.}	inf	37.	45.6621	{32.5176, 57.7373}	30.0063	0.108684	newly	32.	37.92	{27., 50.}	26.125	0.099459	Reff	0.99205	1.08596	{1.06608, 1.10407}	9.46577	ar	400.	507.82	{434., 593.}	27.67
2	R0	{1.33231, 10.5259, 2.}	{1.30842, 3.57722, 0.07}	{{1.24751, 3.07157, 0.}, {1.44762, 4.00571, 0.}}	{6.23964, 66.0151, 98.5}	inf	32.	16.1664	{5.46413, 25.9018}	49.6495	0.36923	newly	17.	25.07	{6., 43.}	72.7647	0.263142	Reff	0.766078	0.772971	{0.729275, 0.862015}	4.93452	ar	467.	451.33	{405., 505.}	8.01499
3	R0	{1.2273, 6.76944, 1.}	{1.15261, 2.53953, 0.03}	{{1.06738, 2.19254, 0.}, {1.25474, 3.07157, 0.}}	{7.80503, 62.4853, 97.}	inf	26.	17.0222	{3.78948, 30.7972}	45.4147	0.333137	newly	25.	42.76	{11., 74.}	96.96	0.316746	Reff	0.996568	0.946087	{0.870363, 1.03649}	7.69004	ar	314.	286.84	{176., 442.}	26.7006
4	R0	{1.41215, 17.5742, 3.}	{1.12156, 2.61419, 0.21}	{{1.06256, 2.19254, 0.}, {1.25474, 3.26445, 1.}}	{20.578, 85.1248, 93.}	inf	25.	12.9519	{3.20643, 24.52}	52.5789	0.41738	newly	9.	31.7	{8., 58.}	263.778	0.51168	Reff	1.19468	0.962171	{0.908881, 1.06458}	19.4619	ar	465.	243.72	{139., 388.}	47.7763
5	R0	{1.42222, 6.15886, 0.1}	{1.36099, 3.21206, 0.02}	{{1.24751, 2.5364, 0.}, {1.47608, 4.06661, 0.}}	{7.90299, 47.8466, 118.}	inf	35.	33.9192	{12.2206, 50.5777}	35.0807	0.181717	newly	37.	62.8	{24., 98.}	84.1081	0.263827	Reff	0.924443	0.8856	{0.799999, 0.970296}	7.33868	ar	490.	469.65	{365., 571.}	13.1694
6	R0	{1.24363, 9.95596, 0.1}	{1.82761, 10.7978, 0.22}	{{1.28596, 3.83841, 0.}, {2.2277, 15.555, 1.}}	{47.1774, 49.524, 278.}	inf	46.	61.9809	{18.383, 97.1457}	64.6743	0.269566	newly	37.	49.08	{28., 64.}	43.6757	0.161427	Reff	1.06828	1.5298	{1.12536, 1.8376}	43.2024	ar	411.	681.73	{370., 858.}	72.0122
7	R0	{1.36478, 5.96056, 1.}	{1.39474, 1.8953, 0.01}	{{1.39162, 1.89883, 0.}, {1.39162, 1.89883, 0.}}	{2.19532, 68.2027, 99.}	inf	43.	34.7234	{3.7402, 71.0673}	53.8947	0.385859	newly	48.	116.38	{16., 222.}	169.792	0.475172	Reff	1.03723	1.07163	{1.01337, 1.11222}	4.3208	ar	484.	440.61	{226., 577.}	19.5062
8	R0	{1.36335, 17.6429, 2.}	{1.27528, 10.8756, 1.07}	{{1.13227, 5.71232, 0.}, {1.36632, 18.7523, 2.}}	{6.83254, 42.507, 49.5}	inf	38.	23.0846	{9.96322, 34.8541}	41.0362	0.271715	newly	14.	18.74	{6., 36.}	67.7143	0.250736	Reff	1.17657	1.1194	{1.01053, 1.19758}	5.76183	ar	484.	375.8	{199., 507.}	25.0992
9	R0	{1.26575, 5.28707, 0.1}	{1.26486, 3.47511, 0.03}	{{1.22544, 3.07157, 0.}, {1.28596, 3.98513, 0.}}	{2.04027, 34.2716, 124.}	inf	37.	20.0137	{6.2533, 33.9846}	49.3861	0.356281	newly	42.	36.35	{10., 59.}	41.119	0.242473	Reff	0.882228	0.903081	{0.868852, 0.926764}	3.01048	ar	472.	382.99	{285., 473.}	21.3835
10	R0	{1.42942, 9.49905, 2.}	{1.58629, 15.6046, 2.79}	{{1.6004, 16.157, 3.}, {1.6004, 16.157, 3.}}	{11.2743, 66.0926, 53.5}	inf	37.	90.377	{77.6653, 107.853}	146.212	0.382916	newly	27.	38.95	{29., 49.}	46.9259	0.162451	Reff	0.86337	0.872625	{0.852054, 0.890087}	1.6583	ar	468.	652.71	{612., 705.}	39.4679
11	R0	{1.46827, 15.0983, 0.1}	{1.26164, 6.92512, 0.92}	{{1.21457, 3.98513, 0.}, {1.3618, 10.1856, 2.}}	{14.0728, 54.1331, 878.}	inf	35.	23.6534	{11.464, 36.3937}	35.869	0.239729	newly	18.	27.36	{13., 50.}	65.8889	0.206592	Reff	1.25831	1.09558	{1.03872, 1.19148}	12.9609	ar	495.	362.39	{168., 492.}	28.4505
12	R0	{1.25751, 13.279, 1.}	{1.17497, 3.13187, 0.38}	{{1.06738, 2.19254, 0.}, {1.3618, 4.98002, 1.}}	{10.8238, 76.4149, 80.}	inf	31.	13.7091	{3.43932, 23.8653}	56.6057	0.465918	newly	18.	32.38	{7., 63.}	105.889	0.340177	Reff	1.09906	1.04684	{0.945524, 1.21499}	10.0968	ar	366.	262.62	{106., 469.}	41.8361
13	R0	{1.3425, 9.16361, 1.}	{1.54816, 15.23, 4.9}	{{1.46004, 13.4586, 5.}, {1.56871, 15.7996, 5.}}	{15.602, 69.0937, 394.}	inf	30.	78.5551	{69.6443, 88.5365}	162.798	0.416625	newly	15.	34.99	{28., 43.}	133.267	0.36109	Reff	0.839063	0.892047	{0.846974, 0.915741}	6.58836	ar	442.	624.65	{581., 678.}	41.3688
14	R0	{1.18936, 6.20583, 0.1}	{1.29021, 4.37942, 0.61}	{{1.24751, 3.98513, 0.}, {1.3618, 5.40945, 2.}}	{8.47944, 29.4452, 636.}	inf	36.	22.2086	{8.60449, 34.5595}	40.5212	0.278217	newly	30.	33.43	{14., 57.}	42.7	0.189755	Reff	0.848014	0.93761	{0.910424, 0.975156}	10.5654	ar	382.	389.02	{291., 478.}	15.8115
15	R0	{1.27137, 9.78868, 0.1}	{1.25181, 3.45029, 0.08}	{{1.21159, 3.07157, 0.}, {1.28596, 3.98513, 0.}}	{2.82904, 64.7522, 164.}	inf	42.	20.0044	{5.41783, 35.678}	53.2369	0.412022	newly	20.	38.16	{12., 68.}	106.	0.302377	Reff	0.943357	0.95632	{0.920843, 0.99349}	3.04982	ar	496.	337.91	{243., 448.}	32.0585
16	R0	{1.23102, 13.6452, 1.}	{1.49526, 16.0412, 3.}	{{1.45513, 15.3398, 3.}, {1.5102, 16.3861, 3.}}	{21.4649, 18.3365, 200.}	inf	28.	46.3226	{34.8904, 54.8719}	65.8168	0.206994	newly	7.	21.1	{14., 30.}	201.429	0.460368	Reff	0.984816	1.16865	{1.13614, 1.19248}	18.667	ar	445.	550.72	{463., 631.}	24.2517
17	R0	{1.27213, 13.7452, 3.}	{1.53795, 11.8849, 1.05}	{{1.21937, 6.0993, 0.}, {1.85141, 18.8228, 2.}}	{23.3807, 34.1002, 65.}	inf	25.	37.4157	{18.3991, 57.3067}	63.2982	0.208469	newly	15.	24.52	{15., 35.}	66.5333	0.205303	Reff	1.11566	1.32644	{1.07486, 1.57826}	21.1713	ar	367.	551.2	{277., 764.}	59.3569
18	R0	{1.25318, 11.2151, 0.1}	{1.29893, 6.76219, 1.44}	{{1.24271, 4.98002, 1.}, {1.3618, 9.41429, 2.}}	{4.1026, 39.7046, 1348.}	inf	34.	21.2356	{9.66397, 31.659}	38.7503	0.253886	newly	18.	23.47	{13., 38.}	47.3889	0.171159	Reff	0.988759	1.04133	{1.00088, 1.08995}	5.35882	ar	416.	369.95	{226., 485.}	19.5601
19	R0	{1.46006, 8.11496, 0.1}	{1.28914, 3.89934, 0.13}	{{1.25474, 3.26445, 0.}, {1.3618, 4.21286, 1.}}	{11.7286, 51.9487, 208.}	inf	40.	24.4134	{9.33168, 39.512}	41.9266	0.29446	newly	35.	38.8	{13., 61.}	41.8286	0.202871	Reff	0.995761	0.899179	{0.863004, 0.925711}	9.81028	ar	496.	403.31	{321., 478.}	19.5585
20	R0	{1.14273, 6.71261, 0.1}	{1.51688, 12.6792, 2.14}	{{1.28596, 3.98513, 0.}, {1.6004, 16.157, 3.}}	{32.7416, 110.039, 2096.}	inf	37.	75.7749	{20.0582, 105.229}	123.664	0.364507	newly	33.	43.	{30., 54.}	38.4848	0.143027	Reff	0.861618	1.08077	{0.985107, 1.12854}	25.4352	ar	362.	583.46	{341., 698.}	65.2597
21	R0	{1.42432, 8.23905, 1.}	{1.24015, 2.67429, 0.}	{{1.22544, 2.5364, 0.}, {1.24751, 3.07157, 0.}}	{12.9301, 67.5413, 100.}	inf	21.	18.8182	{5.03177, 31.5583}	37.5316	0.220905	newly	20.	42.24	{9., 73.}	129.4	0.367261	Reff	1.02409	0.894372	{0.873754, 0.916238}	12.6664	ar	405.	360.33	{280., 450.}	16.9556
22	R0	{1.29386, 17.863, 2.}	{1.67417, 17.6739, 1.93}	{{1.48819, 15.3523, 1.}, {1.75814, 19.4054, 2.}}	{29.3979, 12.8329, 10.5}	inf	30.	43.9045	{23.4585, 54.7267}	55.4931	0.18787	newly	13.	18.67	{10., 27.}	51.7692	0.170275	Reff	1.13601	1.44448	{1.27854, 1.53143}	27.1543	ar	488.	648.29	{492., 756.}	36.8627
23	R0	{1.39274, 13.0167, 5.}	{1.24806, 3.5121, 0.12}	{{1.06738, 2.19254, 0.}, {1.28596, 3.98513, 0.}}	{10.3885, 73.0185, 97.6}	inf	50.	16.8351	{5.95471, 27.7656}	66.3299	0.539299	newly	23.	31.22	{11., 51.}	60.1739	0.22194	Reff	0.997202	0.934871	{0.815584, 0.973358}	6.25056	ar	475.	352.4	{257., 441.}	26.6484
24	R0	{1.32178, 15.8991, 1.}	{1.75544, 19.3911, 2.01}	{{1.75814, 19.4054, 2.}, {1.75814, 19.4054, 2.}}	{32.8088, 21.9635, 101.}	inf	70.	94.2571	{82.1034, 104.2}	35.1907	0.129476	newly	28.	35.67	{29., 43.}	29.1071	0.106841	Reff	1.00059	1.28617	{1.26645, 1.30648}	28.5415	ar	471.	710.72	{662., 762.}	50.896
25	R0	{1.37568, 17.8427, 1.}	{1.32927, 11.4975, 2.2}	{{1.29096, 10.0371, 2.}, {1.48819, 17.9732, 3.}}	{6.97243, 35.8835, 126.}	inf	28.	32.0034	{12.2316, 74.0892}	58.6234	0.224894	newly	9.	18.62	{9., 29.}	110.444	0.293884	Reff	1.11843	1.07356	{1.04644, 1.14138}	4.99361	ar	398.	407.49	{240., 609.}	28.3744
26	R0	{1.20993, 6.4656, 0.1}	{1.4391, 13.8194, 3.21}	{{1.40677, 12.6439, 3.}, {1.54363, 18.3794, 4.}}	{18.9409, 114.727, 3112.}	inf	26.	66.4631	{44.289, 94.3102}	156.843	0.395069	newly	24.	32.97	{25., 39.}	38.625	0.137015	Reff	0.908657	1.02144	{0.99465, 1.0604}	12.4125	ar	345.	550.34	{448., 658.}	60.0696
27	R0	{1.20548, 13.8003, 0.1}	{1.7543, 15.3377, 0.05}	{{1.76949, 15.6127, 0.}, {1.76949, 15.6127, 0.}}	{45.5267, 14.3375, 144.}	inf	33.	58.2504	{49.1071, 68.0487}	78.5973	0.249917	newly	20.	28.46	{21., 36.}	44.4	0.152879	Reff	0.947507	1.33402	{1.32368, 1.36078}	40.7928	ar	350.	680.52	{628., 736.}	94.7829
28	R0	{1.3176, 14.6594, 1.}	{1.82931, 18.6779, 0.99}	{{1.80508, 18.4377, 1.}, {1.86254, 19.1994, 1.}}	{38.8363, 28.5933, 1.}	inf	28.	67.7011	{55.4452, 80.8737}	142.006	0.378407	newly	16.	28.28	{20., 37.}	76.75	0.235815	Reff	1.18979	1.57892	{1.54618, 1.61726}	32.7058	ar	362.	749.51	{698., 801.}	107.047
29	R0	{1.28202, 6.23047, 1.}	{1.53614, 13.5942, 2.12}	{{1.28596, 3.98513, 0.}, {1.63728, 17.5966, 3.}}	{20.378, 135.492, 164.}	inf	21.	81.0868	{16.9439, 115.159}	297.091	0.563288	newly	22.	40.87	{24., 55.}	91.3182	0.280421	Reff	0.937157	1.02436	{0.922734, 1.07575}	10.0374	ar	391.	599.54	{377., 721.}	57.509
30	R0	{1.28177, 8.83252, 1.}	{1.33686, 6.13387, 1.48}	{{1.27231, 4.98002, 0.}, {1.38815, 6.88118, 2.}}	{4.94585, 30.5535, 102.}	inf	41.	33.6696	{19.7663, 50.0609}	28.1088	0.161309	newly	27.	38.42	{21., 55.}	51.1852	0.179416	Reff	0.9562	1.00685	{0.960574, 1.04249}	5.48385	ar	427.	446.34	{331., 542.}	16.7494
31	R0	{1.35886, 5.97708, 0.1}	{1.9588, 15.6335, 1.97}	{{1.93288, 15.3647, 2.}, {2.05052, 16.7932, 2.}}	{44.1502, 161.557, 1870.}	inf	33.	135.652	{119., 156.42}	311.066	0.609522	newly	34.	60.24	{51., 70.}	77.1765	0.244482	Reff	0.841134	1.01005	{0.98692, 1.03755}	20.0823	ar	491.	769.67	{725., 816.}	56.7556
32	R0	{1.27601, 17.9804, 3.}	{1.3745, 13.7762, 1.01}	{{1.21457, 10.1856, 1.}, {1.45613, 15.876, 1.}}	{10.7659, 23.3823, 66.3333}	inf	45.	51.4651	{17.9313, 73.6362}	49.3368	0.222935	newly	24.	26.29	{11., 37.}	34.5417	0.155923	Reff	1.09737	1.1708	{1.06917, 1.2313}	8.06512	ar	445.	515.85	{339., 633.}	29.6157
33	R0	{1.3368, 11.3235, 0.1}	{1.42161, 8.9546, 0.13}	{{1.38427, 8.35044, 0.}, {1.39882, 8.35044, 0.}}	{6.42067, 29.1898, 216.}	inf	59.	50.2983	{33.3838, 63.1038}	31.7418	0.148464	newly	32.	39.2	{28., 54.}	29.375	0.106639	Reff	0.974527	1.04594	{1.01315, 1.04989}	7.32773	ar	417.	511.03	{414., 587.}	24.6691
34	R0	{1.21021, 11.3635, 2.}	{1.19698, 4.09151, 0.36}	{{1.08063, 3.02112, 0.}, {1.28596, 5.77111, 1.}}	{5.45642, 63.9943, 83.}	inf	17.	12.091	{2.72008, 21.7934}	46.0883	0.311282	newly	9.	22.53	{5., 50.}	169.667	0.379965	Reff	1.00689	1.00171	{0.9039, 1.07242}	5.51996	ar	327.	265.49	{161., 402.}	31.1407
35	R0	{1.53382, 10.8866, 0.1}	{1.79072, 17.6604, 1.77}	{{1.42801, 9.02643, 1.}, {1.8189, 18.8228, 2.}}	{18.4218, 66.7664, 1682.}	inf	51.	114.417	{53.6311, 134.94}	126.079	0.341676	newly	30.	46.42	{36., 60.}	54.8667	0.181344	Reff	1.1565	1.23288	{1.09374, 1.27408}	8.21893	ar	476.	729.48	{604., 786.}	53.3361
36	R0	{1.20423, 6.83844, 0.1}	{1.93743, 15.7796, 1.97}	{{1.93882, 15.8893, 2.}, {1.93882, 15.8893, 2.}}	{61.0904, 133.124, 1872.}	inf	51.	128.577	{111.306, 141.354}	153.206	0.396858	newly	46.	58.57	{48., 71.}	28.0652	0.103577	Reff	0.814059	1.15632	{1.1341, 1.19047}	42.2274	ar	416.	767.03	{726., 806.}	84.3822
37	R0	{1.43629, 10.1548, 3.}	{1.29255, 4.65244, 0.58}	{{1.22544, 3.26445, 0.}, {1.31748, 10.8592, 4.}}	{10.4216, 55.8495, 88.6667}	inf	42.	23.2166	{7.74662, 36.28}	45.7578	0.330227	newly	33.	38.22	{14., 66.}	52.303	0.226298	Reff	1.10594	1.01941	{0.976289, 1.0583}	8.44778	ar	367.	355.77	{227., 463.}	20.6567
38	R0	{1.37504, 8.93428, 1.}	{1.56687, 8.55434, 1.1}	{{1.22544, 2.5364, 0.}, {2.1968, 19.9579, 3.}}	{23.3624, 78.1604, 138.}	inf	75.	76.1126	{11.5267, 178.384}	78.5352	0.417922	newly	60.	56.68	{22., 84.}	31.0667	0.168571	Reff	0.897901	0.996933	{0.849503, 1.21588}	14.1528	ar	483.	547.72	{320., 838.}	36.3437
39	R0	{1.51637, 9.36742, 1.}	{1.32756, 4.67698, 0.75}	{{1.28596, 3.98513, 0.}, {1.3618, 4.98002, 2.}}	{12.5512, 51.3737, 61.}	inf	30.	24.108	{9.2193, 39.5518}	35.2744	0.219697	newly	16.	33.54	{13., 53.}	121.25	0.335944	Reff	1.019	0.899741	{0.864197, 0.934691}	11.7435	ar	441.	417.08	{334., 505.}	12.0181
40	R0	{1.23658, 9.82869, 1.}	{1.27608, 3.25343, 0.}	{{1.22544, 2.5364, 0.}, {1.44762, 3.98513, 0.}}	{3.99048, 66.8986, 100.}	inf	71.	26.7116	{10.7304, 45.1657}	62.378	0.502389	newly	51.	53.23	{18., 86.}	39.4314	0.204154	Reff	0.868079	0.952254	{0.905972, 1.06496}	9.84301	ar	414.	387.	{283., 502.}	18.5362
41	R0	{1.24247, 5.4242, 0.1}	{1.28649, 4.33422, 0.81}	{{1.06738, 2.19254, 0.}, {1.57414, 11.6931, 4.}}	{8.33303, 67.2597, 870.}	inf	30.	31.5381	{5.89263, 78.3217}	70.6381	0.359235	newly	38.	49.76	{17., 85.}	59.2105	0.233482	Reff	0.956702	0.985203	{0.842009, 1.13158}	7.48984	ar	345.	395.25	{225., 634.}	35.2203
42	R0	{1.28208, 4.40223, 0.1}	{1.76772, 10.9304, 1.06}	{{1.28596, 3.98513, 0.}, {2.29738, 19.0355, 2.}}	{38.5462, 160.002, 1050.}	inf	25.	89.569	{20.401, 160.353}	269.026	0.485268	newly	44.	58.21	{34., 80.}	45.1591	0.176319	Reff	0.951303	1.17016	{0.926757, 1.42821}	24.3634	ar	339.	633.45	{368., 866.}	88.8407
43	R0	{1.56773, 6.50474, 0.1}	{1.33154, 3.37916, 0.}	{{1.24751, 2.5364, 0.}, {1.47488, 4.00571, 0.}}	{15.066, 48.0508, 100.}	inf	43.	33.3276	{14.9166, 49.0477}	31.6981	0.196738	newly	49.	57.4	{24., 89.}	41.5102	0.17706	Reff	0.981399	0.846047	{0.779276, 0.928102}	13.8289	ar	498.	473.45	{402., 550.}	10.3353
44	R0	{1.10489, 6.01941, 1.}	{1.23751, 3.02962, 0.}	{{1.22544, 2.5364, 0.}, {1.25474, 3.26445, 0.}}	{12.275, 49.6692, 100.}	inf	33.	22.1326	{5.75265, 37.5764}	39.723	0.295661	newly	35.	44.74	{12., 80.}	58.2857	0.248677	Reff	0.79994	0.909335	{0.88542, 0.939261}	13.7967	ar	346.	369.98	{274., 476.}	19.0925
45	R0	{1.25888, 14.1926, 1.}	{1.30908, 4.42207, 0.46}	{{1.28596, 3.98513, 0.}, {1.3618, 4.98002, 2.}}	{3.98765, 69.3369, 80.}	inf	65.	21.6025	{6.05061, 35.6453}	66.8881	0.560495	newly	24.	31.78	{8., 55.}	62.5833	0.24419	Reff	0.815754	0.904737	{0.872029, 0.934993}	10.9081	ar	471.	407.51	{299., 505.}	18.2739
46	R0	{1.45292, 16.616, 2.}	{1.67803, 13.1781, 0.82}	{{1.39882, 8.26611, 0.}, {1.85141, 16.1198, 1.}}	{17.9297, 20.9559, 60.}	inf	45.	56.5254	{28.2886, 77.7316}	43.0775	0.181689	newly	23.	32.73	{22., 45.}	47.6087	0.167936	Reff	1.20738	1.37245	{1.17512, 1.51275}	15.3573	ar	494.	637.45	{450., 746.}	36.6822
47	R0	{1.22528, 16.6033, 0.1}	{1.77998, 18.0648, 1.}	{{1.66003, 16.3716, 1.}, {1.86254, 19.1994, 1.}}	{45.2714, 9.58417, 900.}	inf	28.	49.1794	{37.9968, 58.3633}	75.8468	0.239881	newly	12.	21.41	{14., 29.}	78.5833	0.236259	Reff	1.1101	1.57492	{1.46301, 1.64815}	41.8715	ar	368.	722.62	{660., 775.}	96.3641
48	R0	{1.1669, 6.92715, 0.1}	{1.56495, 19.9037, 5.}	{{1.56495, 19.9037, 5.}, {1.56495, 19.9037, 5.}}	{34.112, 187.329, 4900.}	inf	37.	84.2878	{74.854, 92.7567}	127.805	0.355783	newly	38.	30.57	{24., 39.}	21.1842	0.10757	Reff	0.839001	1.0512	{1.03712, 1.06581}	25.2916	ar	416.	631.38	{587., 671.}	51.774
49	R0	{1.32749, 16.0887, 3.}	{1.44566, 10.8066, 0.09}	{{1.45954, 10.9674, 0.}, {1.45954, 10.9674, 0.}}	{9.43724, 32.8311, 97.}	inf	27.	29.4762	{19.6427, 40.8188}	23.9551	0.102617	newly	11.	20.8	{13., 29.}	91.4545	0.268483	Reff	1.17085	1.27138	{1.26112, 1.29498}	9.06155	ar	353.	524.2	{429., 604.}	49.898
50	R0	{1.29636, 15.2609, 0.1}	{1.57905, 15.1605, 1.97}	{{1.34829, 9.33397, 2.}, {1.73196, 19.1609, 2.}}	{21.9591, 31.8362, 1874.}	inf	42.	55.1104	{36.7405, 69.3546}	39.5167	0.162301	newly	19.	26.78	{18., 37.}	46.8421	0.17268	Reff	0.917823	1.09523	{0.941085, 1.20089}	19.3704	ar	470.	582.27	{397., 706.}	30.1936
